# How Can Digital Financial Inclusion Promote High-Quality Agricultural Development? The Multiple-Mediation Model Research

**DOI:** 10.3390/ijerph20043311

**Published:** 2023-02-14

**Authors:** Hua Zhang, Ying Li, Hanxiaoxue Sun, Xiaohui Wang

**Affiliations:** 1 Sunwah International Business School, Faculty of Economics, Liaoning University, Shenyang 110136, China; 2 Business School, Faculty of Economics, Liaoning University, Shenyang 100136, China; 3 School of Public Management, Faculty of Economics, Liaoning University, Shenyang 100136, China

**Keywords:** digital financial inclusion, high-quality agricultural development, farmland transfer, farmland mechanization level

## Abstract

In order to balance the relationship between economics, society and environment, the Chinese government has stated that China’s economy should shift from high-speed growth to high-quality development. Since agriculture is the foundation of the national economy, high-quality development of agriculture plays significant roles in the achievement of food security, social stability and environmental sustainability in China. In practice, the expansion of digital financial inclusion (DFI) seems to provide valuable opportunities for the development of high-quality agriculture. Nevertheless, in theory, the extant literature ignores exploration of the close relationships between DFI and high-quality agricultural development (HQAD). Hence, using Chinese provincial panel data from 2011 to 2020 and structural equation model (SEM) in STATA 16.0, this paper attempts to investigate whether and how DFI can enhance HQAD. Analysis reveals that (1) DFI can directly promote HQAD; (2) DFI can indirectly facilitate HQAD through the mediator of farmland transfer (FLT); (3) DFI can indirectly promote HQAD through the mediator of farmland mechanization level (FML); (4) compared with the benefits brought by “high-mechanization”, the benefits brought by “large-scale” farmland transfer policies are much greater. To our knowledge, our research is one of the first to investigate the direct and indirect effecting mechanisms of DFI’s influence on HQAD from the perspectives of farmland scale and farmland technology.

## 1. Introduction

In 2021, the COP 26 United Nations Climate Change Conference held in Glasgow emphasized a crucial time mode for mankind’s positive actions and demonstrated that the next 10 years will be a vital last window. If humans fail to take positive actions to address climate change, the Earth will face destructive natural disasters, such as sea level rise and species decline [1]. In such conditions, to balance the relationship between economics, society and environment, the Chinese government proposed that China’s economy should shift from a stage of high-speed growth to a stage of high-quality development, which means that the economy is not only pursuing speed and scale but also quality and efficiency [2]. Agriculture, as the foundation of national economy, plays a significant role in food security, social stability, labor employment and ecological protection [3,4]. The HQAD, as a significant component of Chinese high-quality economic development, has aroused wide concern among Chinese government and the public. HQAD refers to the new driving force: efficient growth, industrial system integration and sustainable development of agriculture [5]. It focuses on a framework of “innovation, coordination, green, openness and sharing” for agricultural development [2].

**Figure 1:**
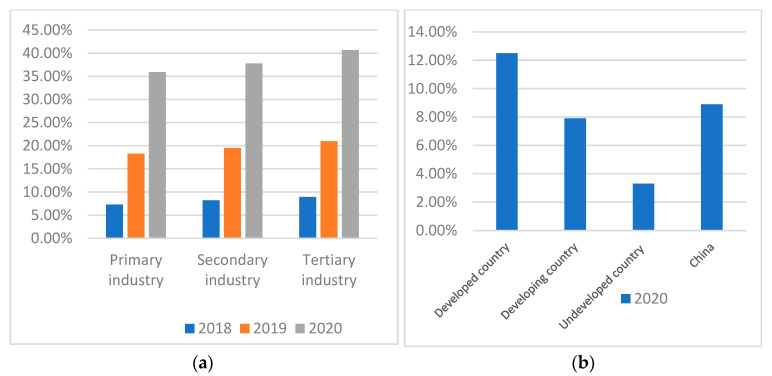
(**a**). The penetration rate of the digital economy in three industries from 2018 to 2020; (**b**). The penetration rate of the digital economy in the agricultural industry of different types of countries in 2020. (Resources: www.iresearch.com.cn; http://www.caict.ac.cn/, accessed on 10 September 2022).

The rapid development of the digital economy seems to provide new opportunities for the HQAD in China [6,7]. Referring to the Ire Research and China Academy of Information and Communications Technology, the penetration rate of the digital economy in the Chinese primary industry has gradually grown from 2018 to 2020 (Figure 1a). Nevertheless, the penetration rate in primary industry (agricultural industry) in China is much lower than that of secondary and tertiary industries (Figure 1a) and that of most developed countries in 2020 (Figure 1b). It is crucial to accelerate the driving effects of the digital economy in agricultural development, wherein DFI, characterized in form by digitalization and inclusiveness [8], can provide significant financial guarantees for high-quality development of Chinese agriculture. However, in theory, research focusing on the relationship between DFI and HQAD is still in the initial stage. The extant literature has verified that DFI can promote agricultural eco-efficiency [9], protect the agricultural supply chain [10], improve agricultural green total factor productivity [11], mitigate farmers’ vulnerability to poverty [12,13] and efficiently integrate rural tertiary industry [14]. Despite that these studies, to some extent, provide some indirect evidence on the relationship between DFI and HQAD, studies directly exploring whether and how DFI can enhance HQAD are scarce.

Farmland is widely regarded as the most fundamental production factor for agricultural development, while fragmented farmland [15,16] and a low mechanization level [17,18] are considered as the main obstacles to agricultural development in China. In the circumstances, on the one hand, the Chinese government continuously encourages the policies of FLT in order to solve the problems of land fragmentation and achieve large-scale operations and farmland protection [19]. On the other hand, the Chinese government repeatedly advocates the introduction of machinery in farming, so as to improve efficiency and achieve high-mechanization [20]. Both FLT and FML seem to be significant promoters of high-quality agricultural development. However, the extant literature ignores exploration of the effects of FLT and FML played out within the relationship between DFI and HQAD.

To address these gaps, this paper attempts to investigate the following problems. First, can DFI directly facilitate HQAD; second, how can DFI indirectly improve HQAD through the mediator of FLT; third, how can DFI indirectly enhance HQAD through the mediator of FML; fourth, is there any difference between the mediating effects emerging from the two mediators within the mechanism.

Based on the provincial panel data in China from 2011 to 2020 and the SEM analysis in STATA 16.0, our research contributes to the extant literature in the following aspects. First, the extant literature ignores exploration of the direct relationship between DFI and HQAD, while our research provides empirical evidence of the positive effects of DFI on HQAD. Second, our research is among the first to identify the positive mediating effects played by FLT and FML on the path of DFI’s effect on HQAD. Third, through the comparison of the difference between the mediating effects, our paper conducts an in-depth analysis of the difference between scale benefits and technology benefits in HQAD.

The remainder of the paper is structured as follows. In the Section 2, we focus on the literature review and hypothesis development. Then, we introduce the data sources, the research method, the constructed model and the measurements of variables. We report the results and relative analyses. Finally, we demonstrate our conclusions, contributions and future research directions in the Section 5.

## 2. Literature Review and Hypothesis Development

### 2.1. Literature Review

There have been some studies focusing on the measurements and determinants of HQAD, which provide a significant basis for our research. Cui et al. [2] and Qin et al. [4] divided HQAD into five dimensions, that is, innovation, coordination, green, openness and sharing, and calculated the index using the unexpected output super-efficiency SBM model, TOPSIS model and entropy method. Lu et al. [5] divided HQAD into four dimensions, new driving power, efficiency growth, industrial system integration and sustainable development and further measured it using the projection pursuit model. Wang et al. [21] described HQAD in four directions, that is, high quality, high yield and high efficiency, high-efficiency agriculture, modern agricultural management systems and green sustainable development, and measured the index using the entropy method. There is also some research using the DEA-SBM model to measure HQAD and the indicators consist of input indicators, desirable output indicators and undesirable output indicators [22]. Besides, the extant literature has verified that farmland recessive morphology transition involving the smooth transition of farmland property rights, input structure, quality and function [5], along with agricultural opening-up [21], are positively related to HQAD. Factor mismatch is negatively related to HQAD [4]. These studies are conducive to identifying the measurements, indicators and antecedent variables of HQAD.

Although the extant literature ignores direct exploration of the relationship between DFI and HQAD, a series of studies focusing on the positive effects of DFI in influencing agricultural development provide the theoretical foundations for our research. First, scholars have identified that DFI is conducive to the enhancement of agricultural production. Specifically, Fang and Zhang [10] empirically verified that 1% increase in the DFI can increase agricultural trade by approximately 1.6%, which means that DFI plays a significant role in protecting the agricultural supply chain. Ge et al. [14] pointed out that DFI can create a healthy rural financial ecological environment, in order to integrate the rural tertiary industries. The extant literature also verified that the development of DFI can significantly increase Chinese farmers’ willingness to adopt agricultural technology in the production process [23]. Second, scholars also verified that DFI can efficiently improve the agricultural ecological environment. For instance, Ma and Li [9] and Guo et al. [24] proposed that DFI can dramatically improve agricultural eco-efficiency and green development. Hong et al. [11] and Gao et al. [25] empirically verified that DFI significantly improves China’s agricultural green total factor productivity, respectively, through the mediating effects of agricultural structure optimization and green technology improvement. DFI is also positively related to agricultural carbon emission performance [26]. Third, there are some studies identifying that DFI can improve the incomes and living standards of rural residents. For instance, Wang and He [12] and Wang and Fu [13] both verified that DFI is conducive to the reduction of the likelihood of poverty for farmers in the future. Accordingly, DFI can contribute to the development of agricultural industry, rural areas and rural residents.

Farmland is the most fundamental production factor for agricultural development. The Chinese government repeatedly encourages the implementation of FLT policies and the enhancement of FML, so as to achieve “large-scale” and “high-mechanization” agricultural production. There have been many studies focusing on the antecedent variables and consequence variables of FLT and farmland mechanization, which further provide the theoretical basis for our research. In terms of FLT, scholars have pointed out that rural residents moving out to urban areas for employment [27], the establishment of large-scale farm enterprises [28], land tenure fragmentation [29] and DFI [30,31] are all drivers of FLT. Furthermore, FLT is conducive to increasing cultivated land green utilization efficiency [32,33] and improving farmland protection techniques and the sustainable development of agriculture [19]. In terms of the mechanization level of farmland, the extant literature pointed out that the agricultural equipment level, the level of economic development, demographic factors, benefit factors [34] and socioeconomic characteristics, available technology and government policies [35] are positively related to the mechanization level of farmland, while small farm size and fragmentation of holdings [36] are negatively related. Further, the level of mechanization has a significant positive impact on the cost, output value, income and return rate of all types of crops [37], along with the increase of grain yield and farmers’ income [38]. These studies provide evidence regarding FLT and FML as the mediators in the role of DFI in enhancing HQAD.

On the whole, as in the extant literature, this paper uses the TOPSIS model and entropy method to calculate HQAD. Five dimensions, “innovation, coordination, green, openness and sharing” and associated indicators are measured. Different from the extant literature, the paper attempts to explore the direct and indirect relationship between DFI and HQAD from the perspective of farmland utilization.

### 2.2. Hypothesis Development

#### 2.2.1. Digital Financial Inclusion and High-Quality Agricultural Development

HQAD refers to the aspects of “innovation, coordination, green, openness and sharing” in the development of agriculture [2]. DFI, characterized in form by digitalization and inclusiveness [8], can comprehensively promote HQAD. Specifically, first of all, previous studies have shown that financial constraints usually decrease agricultural innovation investments and adversely affect agricultural innovation levels [39,40]. Hence, with the expansion of the coverage breadth of digital financing services, financing constraint problems can be relieved. More rural residents have easy access to loan services and insurance services, which are conducive to obtaining investment capital [41] and reducing investment risks in agricultural innovation activities. Consequently, agricultural innovation level can be improved. Second, DFI can efficiently protect the agricultural supply chain and value chain by financial widening, financial deepening and financial services digitization [10,42], subsequently ensuring stable development of agricultural production. Meanwhile, more high-efficiency production factors can be introduced to agricultural production, which further promotes the coordinating development of primary industry along with secondary and tertiary industries. Third, a large number of studies have verified that DFI is a significant promoter of agricultural green development [9,11,24,25]. To be specific, DFI can promote agricultural industrial structure optimization [11], improve energy utilization efficiency [43] and reduce carbon dioxide emissions [44,45], and consequently promote the green development of agriculture. Fourth, with the expansion of the usage depth of digital financial services, more agricultural product transactions are conducted through digital platforms. Digital platforms can bring open resource advantage, linkage advantage and integration advantage to agricultural development, dramatically expanding trade transaction scope. It promotes globalization by fortifying deeper, broader, and more intricate connections between nations, businesses and individuals [46]. Hence, DFI can efficiently promote the openness level of agriculture. Finally, a large number of studies have verified that DFI can mitigate farmers’ vulnerability to poverty [12,13]. Narrowing the income gaps between rural and urban areas can enhance the sharing level of high-quality agriculture. Based on the above analyses, DFI can improve the aspects of innovation, coordination, green, openness and sharing level in agriculture, consequently promoting HQAD. Hence, the following hypothesis is proposed.

**Hypothesis 1.**  *DFI positively affects HQAD*.

#### 2.2.2. The Mediating Effects of Farmland Transfer

##### Digital Financial Inclusion and Farmland Transfer

With the development of DFI, the coverage breadth, usage depth and digitization level of financial services will increase, which, in turn, will promote FLT. First, the expansion of the coverage breadth broadens the areas and populations that are available for digital financial services [47]. Digital financial services can efficiently alleviate the problems of information asymmetry, reduce the transaction costs and promote the optimization of capital allocation [48]. To be more specific, both the transfers’ and the transferees’ information on assets and credit are easily available online, which strengthens the trustworthiness and cooperation between the two sides. Meanwhile, convenient and low-cost financial services reduce the total transaction costs; hence, the willingness of conducting FLT transactions of both parties will increase [49]. Second, along with the deepening of the usage of digital finance, more non-agricultural entrepreneurship and employment opportunities are available for rural residents, which further promotes farmland transfer [13]. Specifically, since rural residents have easy access to funds at reasonable interest rates without collateral assets, they are more likely to make off-farm investments and start their own business [50]. Besides, it has been verified that the development of DFI can efficiently drive regional economic growth, especially the development of small and medium-sized enterprises [51]; thereby, more employment opportunities can be provided to rural residents. With the transfer of labor from agriculture to non-agriculture, FLT activities will be more frequent [27]. Third, with the development of the digitalization level of financial services, agricultural modernization can be promoted [52]. Digital technologies such as big data, cloud computing and artificial intelligence can be introduced to agricultural production. This is conducive to the optimization of agricultural structure, so as to improve agricultural incomes, which, to some extent, attracts some farmers to buy farmland in order to expand the farm-land operating scale [11]. Consequently, FLT activities can be enhanced. On a basis of these analyses, we propose the following hypothesis:

**Hypothesis 2.**  *DFI positively affects FLT*.

##### Farmland Transfer and High-Quality Agricultural Development

FLT can drive HQAD in the following aspects. First of all, FLT is always accompanied by the transferring of managing rights from individual farmers to professional operators and groups. These specialists are more likely to introduce new technologies, which are the significant driving forces of agricultural innovation [53]. With the participation of professional groups and the introduction of advanced technologies, the agricultural productivity will dramatically increase [54], which further promotes the coordinated development of primary industry with secondary and tertiary industries. The specialists usually have a good knowledge of the precise utilization of fertilizer and pesticides, subsequently reducing the emissions of carbon and other pollution and promoting the green development of agriculture [55]. Besides, it has been verified that the formal signing of the FLT contract is an efficient way to stabilize long-term farmland management rights, which drives the operators to improve the awareness of land protection and sustainable development [19]. On the whole, FLT, accompanied by the transferring of management rights from low-efficiency operators to high-efficiency operators, can promote innovation, coordination and green agricultural development.

In addition, as Lu et al. [19] and Zhou et al. [33] pointed out, FLT can solve the problems of land fragmentation and expand agricultural operation scale. Hummels and Klenow [56] proposed that large economies export more in absolute terms than small ones. Large-scale operation is conducive to the development of agricultural product exports and foreign trade, for the reason that they usually have financial and information advantages and are easily accessible to policy support. Meanwhile, large-scale operations are more likely to attract foreign investments and support than smaller ones. Besides, it has been recognized that FLT can connect small plots of cultivated land and subsequently enlarge the area of cultivated land [33]. Overall agricultural productivity and outputs could be significantly increased; hence, income gaps between rural and urban areas can be reduced. With the increase in farmers’ incomes, rural education and health, living standards can grow as well [57,58]. To sum up, FLT can promote the development of openness and sharing in agriculture by expanding the operating scale. Thereby, we propose the following hypothesis:

**Hypothesis 3.**  *FLT positively affects HQAD. Combining Hypotheses 1–3, we further propose:*

**Hypothesis 4.**  *DFI positively affects HQAD through the mediating effects of FLT.*

#### 2.2.3. The Mediating Effects of Farmland Mechanization Level

##### Digital Financial Inclusion and Farmland Mechanization Level

With the development of DFI, the farmland mechanization level can be greatly improved. First of all, according to Zhou et al. [23], rural residents’ willingness to adopt agricultural technologies is affected by the availability of funds. Since traditional institutions have difficulty offering adequate financial products and services to rural residents, rural residents are either short of mortgage collateral or faced with the risk of bankruptcy after the loss of mortgage collateral. Rural residents’ low loan willingness forces them to choose traditional production methods instead of high-technology mechanization production [59]. With the expansion of the coverage breadth of digital finance, more rural residents can fund support due to lower loan thresholds and restrictions. This is conductive to the enhancement of FML. Second, as mentioned above, along with the deepening of the usage of digital finance, more non-agricultural entrepreneurship and employment opportunities can be provided to rural residents [13]. To some extent, this drives the shift of labour from agriculture to non-agriculture. With a shrinking rural labor force, agricultural mechanization, as an alternative production factor, will be widely applied in agricultural production and FML can be improved. Third, DFI is the cross-border integration of financial inclusion and digital technology. With the increase in the digitalization level of financial services, it is easier to collect various information from farmers’ networks, integrate scattered information into integrated and valuable financial data, and scientifically calculate and identify suitable financing profiles for farmers [60]. Further, with the reduction of farmers’ financing risk and the improvement of insurance service quality, farmers are more likely to introduce high-technology mechanization into production [23]. Accordingly, the following hypothesis is proposed:

**Hypothesis 5.**  *DFI positively affects FML.*

##### Farmland Mechanization Level and High-Quality Agricultural Development

The improvement of FML can comprehensively promote HQAD through the improvement of the various aspects of agricultural innovation, coordination, green, openness and sharing level. Specifically, according to Mehta et al. [61], modern agricultural production is transferring from highly labor intensive to smart agriculture, based on machines and digital technologies. Smart agriculture consists of the applications of sensors, controllers, the Internet of Things (IoT), artificial intelligence (AI) and robots in farmland utilization and agricultural production. According to Maria et al. [62], the new digital technologies are significant drivers of agricultural innovation. Furthermore, smart agriculture usually combines a precision farm with management tools (e.g., GPS, GNSS, DSS, GIS, etc.), data solutions (e.g., data LoT, tech empowered tools) and end user applications (e.g., mobile, platforms, machines) [61]. With the application of smart machines, the production efficiency and quality of farmland can be improved, which, in turn, promotes the coordination development of primary industry with secondary and tertiary industries. Besides, smart mechanization is conducive to savings in inputs, for instance, seeds, water, energy, pesticides and fertilizers, dramatically enhancing agricultural green and sustainable development [63]. In addition, FML can promote the export of agricultural goods and services. Referring to Mehta et al. [61] and Yi et al. [64], with the improvement of the mechanization level of cultivated land, the productivity and outputs of agricultural products can be dramatically increased. Hence, after satisfying the needs of local residents, surplus agricultural products can be exported to other countries, which promotes the development of the agricultural export trade and improves the level of agricultural openness. Meanwhile, with the increase of agricultural productivity and outputs, income gaps between rural and urban areas can be reduced. With the increase of farmers’ incomes, rural education, health and living standards can also grow up [57,58]. Based on this analysis, FML can improve the aspects of agricultural innovation, coordination, green, openness, and sharing level, which, in turn, facilitates HQAD.

**Hypothesis 6.**  *FML positively affects HQAD. Combining Hypotheses 1, 5 and 6, we further propose the following hypothesis:*

**Hypothesis 7.**  *DFI positively affects HQAD through the mediating effects of FML.*

## 3. Methodology

### 3.1. Research Region and Data Source

Chinese provincial panel data from 2011–2020 were collected to test the seven hypotheses. Among the 34 provincial administrative units in China, Hong Kong, Macao and Taiwan and Tibet are not included due to the unavailability of data.

The measurement indicators of HQAD, FLT and farmland management scale were collected from the China Statistical Yearbook, China Land and Resources Statistical Yearbook, China Rural Statistical Yearbook and Provincial Statistical Yearbook, etc. The interpolation method was used to fill the gaps caused by missing data in each year. The measurement indicators of DFI were collected from the Peking University DFI index.

### 3.2. Measreuments of Variables

#### 3.2.1. Measurement of the Explained Variable

**Figure 2:**
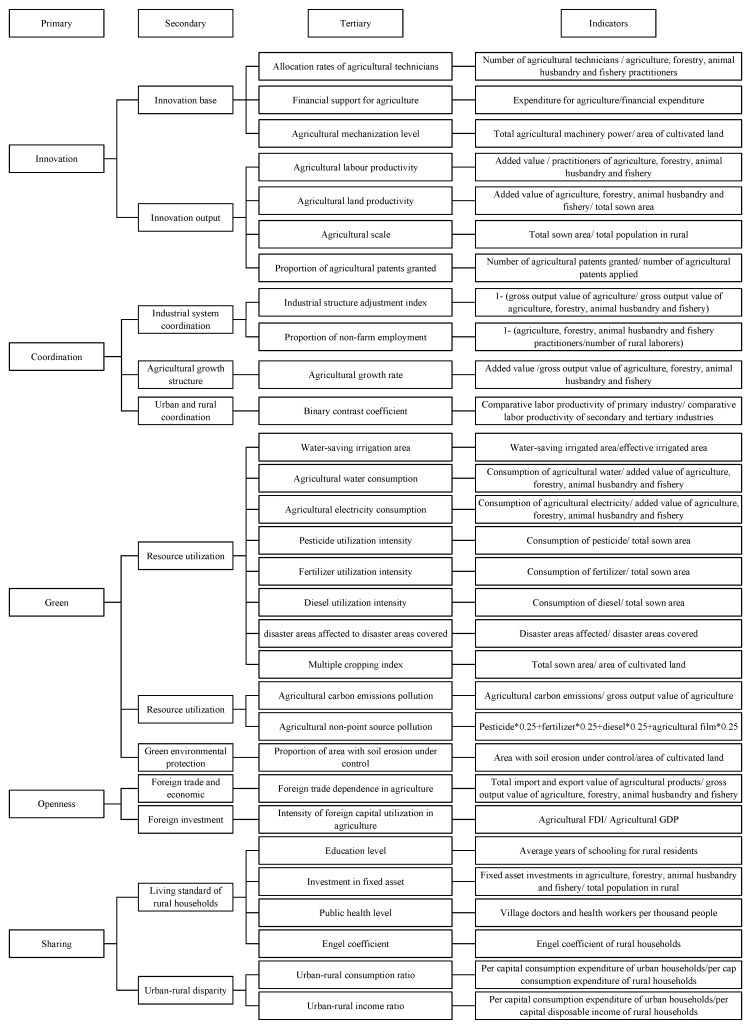
Measurements indicators of high-quality agricultural development.

The explained variable of this study is FLT. According to Zhou et al. [65] and Qin et al. [4], this paper used the entropy weight Technique for Order Preference by Similarity to an Ideal Solution (TOPSIS) method to measure HQAD. The index aggregate consists of five dimensions: innovation, coordination, green, openness, and sharing. The measurement system is shown in Figure 2. The measurement steps are illustrated as follows:

(1) Construction of original evaluation matrix. Supposing the existence of n evaluation objects and m evaluation indexes, the paper sets the original evaluation matrix X for HQAD as follows:
  (1) $$X=\left[\begin{array}{cccc} x_{11} & x_{12} & \cdots & x_{1n}\\ x_{21} & x_{22} & \cdots & x_{2n}\\ \vdots & \vdots & \vdots & \vdots \\ x_{m1} & x_{m2} & \cdots & x_{mn} \end{array}\right]\;$$
(2) Data standardization.
  (2) $$\mathrm{Positive}\;\mathrm{indexes}:\;z_{ij}=\frac{x_{ij}-\min\left(x_{ij}\right)}{\max\left(x_{ij}\right)-\min\left(x_{ij}\right)}$$
  (3) $$\mathrm{Negative}\;\mathrm{indexes}:\;z_{ij}=\frac{\max\left(x_{ij}\right)-x_{ij}}{\max\left(x_{ij}\right)-\min\left(x_{ij}\right)}$$
  (4) $$Z=\left[\begin{array}{cccc} z_{11} & z_{12} & \cdots & z_{1n}\\ z_{21} & z_{22} & \cdots & z_{2n}\\ \vdots & \vdots & \vdots & \vdots \\ z_{m1} & z_{m2} & \cdots & z_{mn} \end{array}\right]$$
  where Z is the standardized matrix.
(3) Calculation of index weights. This paper identifies the weights of the evaluation indexes by the entropy weight method:
  (5) $$e_{i}=-\frac{\sum_{1}^{n}p_{ij}\cdot \ln p_{ij}}{\ln n}$$
  (6) $$w_{i}=\frac{1-e_{i}}{\sum_{1}^{m}\left(1-e_{i}\right)}$$
  where $e_{i}$ represents the entropy value of the i-th index $\left(i=1,2,\cdots ,m\right)$, $w_{i}$ represents the weight of the i-th index $\left(i=1,2,\cdots ,m\right)$ and $p_{ij}=\frac{z_{ij}}{\sum_{1}^{n}z_{ij}}$ represents the calculation of the weight of the i-th index $\left(i=1,2,\cdots ,m\right)$ in year j $\left(j=1,2,\cdots ,n\right)$.
(4) Establishment of weighted normalized evaluation matrix. This paper combines the standardized matrix Z with the index weights $w_{i}$ to establish the weighted normalized evaluation matrix Y:
  (7) $$Y=\left[\begin{array}{cccc} z_{11}w_{1} & z_{12}w_{1} & \cdots & z_{1n}w_{1}\\ z_{21}w_{2} & z_{22}w_{2} & \cdots & z_{2n}w_{2}\\ \vdots & \vdots & \vdots & \vdots \\ z_{m1}w_{m} & z_{m2}w_{m} & \cdots & x_{mn}w_{m} \end{array}\right]=\left[\begin{array}{cccc} y_{11} & y_{12} & \cdots & y_{1n}\\ y_{21} & y_{22} & \cdots & y_{2n}\\ \vdots & \vdots & \vdots & \vdots \\ y_{m1} & y_{m2} & \cdots & y_{mn} \end{array}\right]$$
(5) Determination of positive and negative ideal solutions.
  (8) $$Y^{+}=\left\{\underset{1\leq i\leq m}{\max}y_{ij}|i=1,2,\cdots ,m\right\}=\left\{y_{1}^{+},y_{2}^{+},\cdots ,y_{m}^{+}\right\}$$
  (9) $$Y^{-}=\left\{\underset{1\leq i\leq m}{\min}y_{ij}|i=1,2,\cdots ,m\right\}=\left\{y_{1}^{-},y_{2}^{-},\cdots ,y_{m}^{-}\right\}$$
  where $Y^{+}$ is the positive ideal solution, and $Y^{-}$ is the negative ideal solution.
(6) Calculation of Euclidean distance.
  (10) $$D_{j}^{+}=\sqrt{\overset{m}{\underset{i=1}{\sum }}{\left(y_{i}^{+}-y_{ij}\right)}^{2}}$$
  (11) $$D_{j}^{-}=\sqrt{\overset{m}{\underset{i=1}{\sum }}{\left(y_{i}^{-}-y_{ij}\right)}^{2}}$$
  where $D_{j}^{+}$ represents the Euclidean distance between the positive ideal solution and per evaluation object. $D_{j}^{-}$ represents the Euclidean distance between the negative ideal solution and per evaluation object.
(7) Calculation of closeness.
  (12) $$C_{j}=\frac{D_{j}^{-}}{D_{j}^{+}+D_{j}^{-}}$$
  where $C_{j}$ ranges from [0, 1]. The larger the $C_{j}$ is, the closer HQAD is to the optimal level. $C_{j}=1$ represents that the degree of HQAD is the highest, and $C_{j}=0$ represents that the degree of HQAD is the lowest.

#### 3.2.2. Measurement of the Explanatory Variable

**Figure 3:**
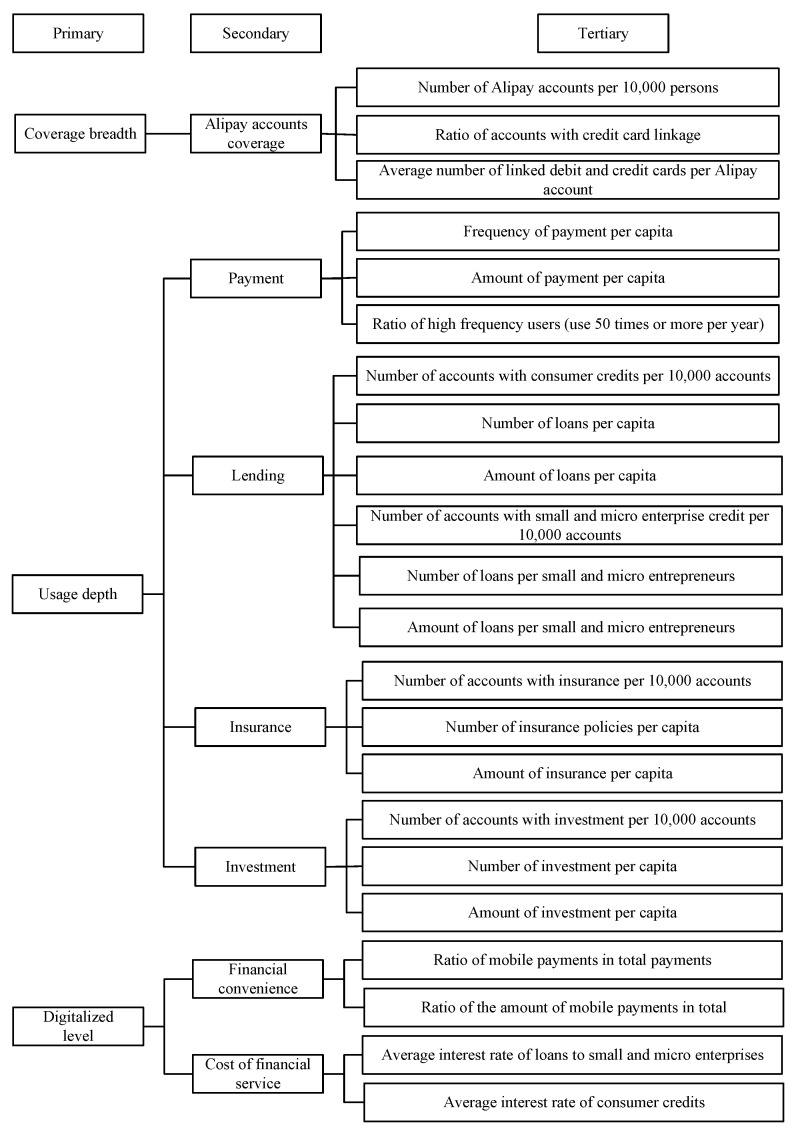
Measurements indicators of DFI.

The explanatory variable of this study is DFI. According to Guo et al. [66], this paper used the Peking University DFI Index of China to measure this. The index aggregate consists of three dimensions, coverage breath, usage depth and digitalized level. The measurement indicators are illustrated in Figure 3.

#### 3.2.3. Measurement of the Mediating Variables

The mediating variable of this study are FLT and FML. Referring to Liu and Liu [67] and Kuang and Peng [68], FLT is measured by the proportion of total area of transferred farmland to total area of contracted farmland of farm households. Referring to Wu et al. [69] and Sun et al. [70], FML is measured by the ratio of total mechanization power of farmland to total area of farmland.

### 3.3. Model Construction 

#### 3.3.1. Models of Main Effects

Based on the relationships between the explanatory variable and the explained variable, this paper constructs the following path model of the main effects

(13) $$hqad_{i,t}=c_{1}dfi_{i,t}+e_{i,t}$$

where *hqad_i,t_* represents the HQAD of the province *i* in the year *t*, *dif_i_*,_*t*_ represents the DFI index of province *i* in the year *t*, *e_i_*,_*t*_ represents the error term and *c*_1_ is the path coefficient of DFI affecting HQAD. If the path coefficient *c*_1_ is significantly positive, H1 will be verified.

#### 3.3.2. Models of Mediating Effects

On the basis of the proposed relationships among the explanatory variable, the mediating variables and the explained variable, this paper constructs the following path model of the mediating effects.

(14) $$\left\{\begin{array}{c} flt_{i,t}=a_{1}dfi_{i,t}+e_{i,t}\\ fml_{i,t}=b_{1}dfi_{i,t}+e_{i,t}\\ hqad_{i,t}=d_{1}flt_{i,t}+d_{2}fml_{i,t}+c_{1}^{‘}dfi_{i,t}+e_{i,t} \end{array}\right.$$

where *flt_i_*,_*t*_ represents FLT ratio of the province *i* in the year *t*, *a*_1_ represents the path coefficient of DFI influencing FLT, *fml_i_*,_*t*_ represents FML of the province *i* in the year *t*, *b*_1_ represents the path coefficient of DFI influencing FML and *d*_1_ *d*_2_ and *c*_1_‘, respectively, represents the path coefficient of FLT, FML and DFI influencing HQAD. If the path coefficient *a*_1_ is significantly positive, H2 will be verified. If the path coefficient *d*_1_ is significantly positive, H3 will be supported. If the mediating path coefficient *a*_1_ *× d*_1_ (*dfi → flt → hqad*) is significantly positive, DFI will positively affect HQAD through the mediator of FLT and H4 will be verified. If the path coefficient *b*_1_ is significantly positive, H5 will be verified. If the path coefficient *d*_2_ is significantly positive, H6 will be supported. If the mediating path coefficient *b*_1_ × *d*_2 (_*dfis → fml → hqad*) is significantly positive, DFI will positively affect HQAD through the mediator of FML and H7 will be verified.

## 4. Results

### 4.1. Descriptive Statistics of Variables and Dimensions

Table 1 illustrates the descriptive statistics of the variables. First, the mean values of the three dimensions of DFI are 196.700, 211.100, and 290.100, respectively; the standard deviations are 96.560, 98.190 and 117.300, respectively. This indicates that the data of the three dimensions of DFI vary dramatically, since the minimum values of the three dimensions are 1.960, 6.760 and 7.580, respectively, revealing that the levels of DFI of some provinces are much lower than the average level. Second, the mean value, standard deviation, minimum value and maximum value of FLT are 0.316, 0.163, 0.034 and 0.911, respectively. This means that the data on FLT vary slightly and the ratios of FLT in most provinces are at relatively low levels. Similarly, The data on FML and HQAD vary slightly and the data in most provinces are at relatively low levels. In addition, the degree of variation based on the mean of HQAD is the greatest, with coefficients of variation of 0.652. On the contrary, the degree of variation based on the mean of FML is the smallest, with coefficients of variation of 0.117.

### 4.2. SEM Results of the Main Effects

**Figure 4:**
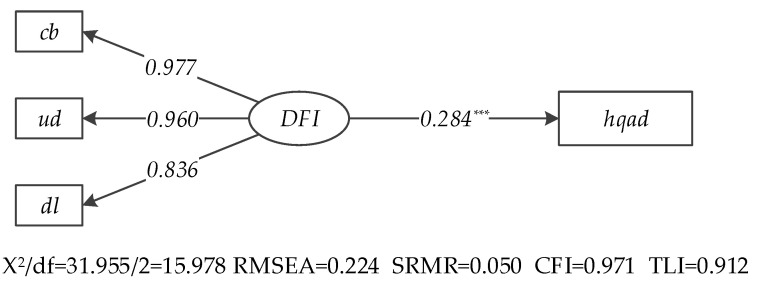
Path diagram and empirical results of main effects (Note: *** represents that the path coefficient is significant at the 1% level).

The SEM results of the main effects are presented in Figure 4 and Table 2. The X^2^/df value is 15.978, the RMSEA value is 0.224 (less than 0.05, unsatisfactory), the SRMR value is 0.050 (less than 0.08), and CFI and TLT are 0.971 and 0.912, respectively (more than 0.9, close to 1). This indicated accepted goodness of fit of the main effect model [71]. The factor loadings of coverage breath, usage depth and digitalized level on DFI are 0.977, 0.960 and 0.836, respectively. Furthermore, the path coefficient of DFI on HQAD is 0.284, passing the significant test at 1% level. DFI is positively related to HQAD and hypothesis 1 is supported. DFI makes it easier for rural residents to obtain loan services and insurance services, which guarantees the rural residents’ fund demands and the sustainable development of agricultural supply chain, and subsequently promotes HQAD.

### 4.3. SEM Results of the Mediating Effects

**Figure 5:**
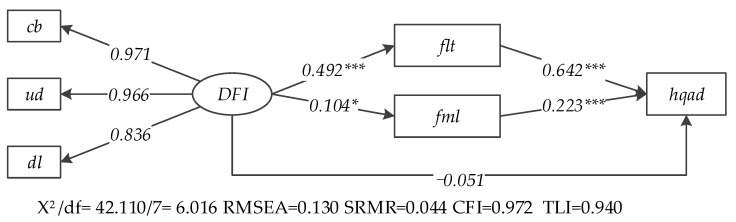
Path diagram and empirical results of mediating effects (Note: *** means the path coefficient is significant at the 1% level; * means the path coefficient is significant at the 10% level).

The SEM results of the multiple mediating effects are illustrated in Figure 5 and Table 3. The X^2^/df value is 6.016, the RMSEA value is 0.130 (larger than 0.05, unsatisfactory) and the SRMR value is 0.044 (smaller than 0.08). CFI and TLT were 0.972 and 0.940, respectively (close to 1). This indicated the accepted goodness of fit of the mediating effect model [71]. The factor loadings of coverage breath, usage depth and digitalized level on DFI are 0.971, 0.966 and 0.836, respectively.

In terms of the mediating effects of FLT, first, the path coefficient of DFI on FLT is 0.492, significant at the 1% significant level. Hence, DFI is positively related to FLT and hypothesis 2 is supported. Second, the path coefficient of FLT on HQAD is 0.642, passing the 1% significant level; hence, FLT is positively related to HQAD and hypothesis 3 is verified. Third, based on the results of the significance of mediating tests (see Table 4), the indirect path coefficient *a*_1_ *× d*_1_ (*dfi → flt → hqad*) is 0.316, passing the significant test at 1% level. This indicates that DFI can positively affect HQAD through the mediating effects of FLT and hypothesis 4 is verified. The development of DFI can alleviate the problems of information asymmetry and reduce the transaction costs of FLT, which improves farmers’ willingness towards FLT. Then, the “large scale” agricultural production brought by FLT will further promote HQAD.

In terms of the mediating effects of FML, first, the path coefficient of DFI on FML is 0.104, significant at the 10% level. Hence, DFI is positively related to FML and hypothesis 5 is supported. Second, the path coefficient of FML on agricultural high-quality development is 0.223, passing the 1% significant level; hence, FML is positively related to HQAD and hypothesis 6 is verified. Third, based on the results of the significance of mediating tests (see Table 4), the indirect path coefficient *b*_1_ × *d*_2_ (*dfi → fml → hqad*) is 0.023, passing the significant test at 1% level. This indicates that DFI can positively affect HQAD through the mediating effects of FML and hypothesis 7 is verified. Thanks to the development of DFI, farmers can obtain more funds to achieve “high-technology” mechanization production, smart agriculture and precision agriculture, which can efficiently promote HQAD.

Furthermore, since the path coefficient of DFI on HQAD is −0.051, not significant. FLT and farmland mechanization play complete mediating effects on the relationship between DFI and HQAD.

### 4.4. Difference Analysis Results of the Mediating Effects

Table 5 illustrates the results of difference tests of mediating path coefficients. In the above analyses, it has been verified that DFI can positively affect HQAD through the increasing FLT and the increasing FML. Furthermore, coefficient difference tests are conducted. The difference coefficient of *a*_1_ × *d*_1_ − *b*_1_ × *d*_2_ is 0.293, significant at 1% level. This indicates that, compared with the promoting effects brought by the increasing FML, the positive effects brought by the increasing FLT are larger within the path of DFI affecting HQAD. Compared with the “high-mechanization” benefits brought by FML, “large-scale” benefits brought by FLT play more significant mediating roles in the path of DFI affecting HQAD.

## 5. Discussion

### 5.1. Discussion of the Empirical Results

This paper empirically investigates whether and how DFI can facilitate HQAD. To verify the proposed hypotheses, this paper collected the Chinese provincial panel data from 2011 to 2020 and used the SEM to conduct the path analyses.

First, DFI is positively related to HQAD, with the path coefficient of +0.284, significant at the 1% level. The empirical results indicate that DFI can directly promote HQAD. With the expansion of DFI, rural residents have easy access to loan services and insurance services. These services increase availability of investment funds and decrease investment risks, contributing to the improvement of agricultural innovation and export activities. Besides, the development of DFI can efficiently protect the agricultural supply chain and improve agricultural yield and productivity. The enhancement of agricultural incomes and profits promotes the coordinated development of primary industry with secondary and tertiary industries and improves the living standards of rural residents. Additionally, DFI can promote agricultural industrial structure optimization, improve energy utilization efficiency and reduce carbon dioxide emissions, which consequently promotes the green development of agriculture. On the whole, DFI directly facilitates agricultural “innovation, coordination, green, openness, and sharing” levels.

Second, DFI can indirectly improve HQAD through the mediator of FLT, with the indirect path coefficient of +0.316, significant at the 1% level. This indicates that DFI can enhance FLT, which, in turn, promotes HQAD. The essence of FLT is the transfer of farmland management rights from individual farmers to professional farmers and groups. The processes are usually accompanied by the expansion of the management scale of farmland. The development of DFI can alleviate the problems of information asymmetry and reduce the transaction costs of FLT, which improves farmers’ willingness towards FLT. Besides, thanks to the availability of funds, farmers have access to off-farm entrepreneurship and employment opportunities, which, to some extent, promote FLT activities. Furthermore, FLT is an efficient way to achieve “large scale” agricultural production. More professional operators and new technologies can be introduced into agricultural production, which is conducive to precise utilization of fertilizer and pesticide, efficient improvement of agricultural productivity and great attraction to foreign investment, etc., consequently promoting HQAD.

Third, DFI also indirectly enhances HQAD through the mediator of FML. The indirect path coefficient is +0.023, significant at the 10% level, indicating that DFI can improve FML, and subsequently promotes HQAD. Capital is an important factor that affects farmland mechanization and modernization level. The traditional institutions have difficulty offering adequate financial products and services to rural residents; hence, rural residents are either short of mortgage collateral or face the risk of bankruptcy when introducing agricultural machinery and equipment. Thanks to the development of DFI, it is easier to collect various information from farmers’ networks, integrate scattered information into integrated and valuable financial data and scientifically calculate and identify suitable financing profiles for farmers. Since farmers can obtain more funds to achieve “high-technology” mechanization production, smart agriculture and precision agriculture can be achieved. New machinery and equipment, such as sensors, controllers, Internet of Things (IoT), artificial intelligence (AI) and robots, can improve the agricultural “innovation, coordination, green, openness, and sharing” level and subsequently promote HQAD.

Finally, compared with “high-mechanization” benefits brought by FML, “large-scale” benefits brought by FLT play more significant mediating roles in the path of DFI affecting HQAD. According to the results of difference analysis tests, the difference coefficient is 0.293, significant at the 1% level, revealing that DFI can dramatically bring “large-scale” advantages via FLT, which, consequently promotes HQAD.

### 5.2. Theoretical Implications

Our research contributes to the extant literature in the following aspects. First of all, the extant literature has verified that DFI can promote agricultural eco-efficiency [9], protect the agricultural supply chain [10], improve agricultural green total factor productivity [11], mitigate farmers’ vulnerability to poverty [12,13] and efficiently integrate rural tertiary industry [14], etc. Nevertheless, research direct exploring the relationship between DFI and HQAD is scarce. Our research theoretically confirms and empirically verifies that DFI can directly facilitate HQAD, providing new evidence on the relationship between DFI and HQAD. Second, the extant literature also verified that the farmland recessive morphology transition involving the smooth transition of farmland property rights, input structure, quality and function [5], and agriculture opening-up [21] are positively related to HQAD. Factor mismatch [4] is negatively related to HQAD. Our research proposes that DFI can positively affect HQAD, which expands the research scope of the determinants of HQAD. Third, another contribution of this study is that it provides a deeper understanding of mechanisms of how DFI can promote HQAD through the mediators of FLT and FML. The extant literature identified that rural residents moving out to urban areas for employment [27], the establishment of large-scale farm enterprises [28], land tenure fragmentation [29] and DFI [30,31] are all drivers of FLT. Furthermore, FLT is conducive to increasing cultivated land green utilization efficiency [32,33] and improving farmland protection techniques and the sustainable development of agriculture [19]. The extant literature also pointed out that the agricultural equipment level, the level of economic development, demographic and benefit factors [34], socioeconomic characteristics, available technology, and government policies [35] are positively related to the mechanization level of farmland, while small farm size and fragmentation of holdings [36] are negatively related to the mechanization level of farmland. Further, the level of mechanization has a significant positive impact on the cost, output value, income and return rate of all types of crops [37] and the increase of grain yield and farmers’ income [38]. Nevertheless, the existing studies ignored investigation of the mediating effects of FLT and farmland mechanization in the relationship between DFI and HQAD. Our research fills these research gaps.

### 5.3. Practical Implications

Our findings also provide some practical guidance First, governments are suggested to vigorously promote DFI, because the expansion of DFI can efficiently facilitate HQAD. Based on the empirical results, specific development methods include the expansion of coverage breadth, usage depth and digitalization level of digital finance. The government, for instance, can reinforce the infrastructure construction of digital technologies to increase equipment coverage in remote areas. Besides, government can reduce the loan interest to promote the usage depth of DFI. Second, government is recommended to encouraging the implementation of FLT policies. For instance, simplifying the procedures and reducing the trade expenses of FLT are some of the efficient ways to promote FLT policies. Through the combination of digital finance and FLT policies, the high-quality development of agriculture can be efficiently achieved. Third, in order to promote FLT, it is necessary for the financial institutions to design and offer diverse portfolios of loans for rural residents to expand the operating scale and introduce agricultural machinery and equipment. In particular, financial institutions are advocated to provide conveniences for green finance to promote HQAD. Fourth, as for agricultural operators, taking advantage of digital finance services such as the digital loan and insurance services can efficiently improve agricultural field and productivity. They are suggested to make use of digital finance to expand the agricultural production scale and improve the agricultural mechanization level to promote agricultural incomes and profits.

### 5.4. Limitations and Future Research Directions

This research has several potential limitations. First, the research data were collected from the mainland of China, which means that the findings might be not valid in the context of other countries. Second, due to the limitations of time and resources, this research only selected FLT and FML as the mediators and other associated variables were not studied. Third, the DFI index was mainly measured by the application situation of Alipay in China. With the development of digital technologies and digital applications, more digital financial tools should be considered and analyzed. These limitations, to some extent, help us to identify future research directions. Future research can replicate this research using the data from other countries to investigate the effect of culture on our model. Future research should explore the mediating roles of other constructs (e.g., farmland management scale, planting structure of farmland, etc.) on the effects of DFI on HQAD. Additionally, more digital financial tools (e.g., WeChat Pay) can be introduced to measure the DFI index.

## 6. Conclusions

The objective of the paper was to fill a significant gap in the literature regarding the impacts of DFI on FLT and FML, which eventually impacts HQAD. This paper collected the Chinese provincial panel data from 2011 to 2020 and used the SEM to conduct the path analysis. The empirical results revealed that DFI can directly improve HQAD. DFI can indirectly enhance HQAD through the multiple mediators of FLT and FML. Compared with the “high-technology” advantages brought by FML, the “large-scale” advantages brought by FLT played more significant roles in the relationship of DFI influencing HQAD. Hence, one of the main contributions of this paper is that it empirically investigated the direct effects of DFI’s influence on HQAD. Another main contribution was its examination of the mediating roles of FLT and FML on the relationships between DFI and HQAD. Based on the research, the government is suggested to reinforce the infrastructure construction of digital finance and encourage the implementation of FLT policies to promote HQAD. Financial institutions are recommended to design and offer diversified portfolios of loans for rural residents to develop agricultural production. Agricultural operators are encouraged to take advantage of digital finance services to expand the agricultural production scale and improve the agricultural mechanization level to increase agricultural incomes and profits.

## Figures and Tables

**Table 1:** Results of descriptive statistics.

Variables	Mean	Standard Deviation	Coefficients of Variation	Minimum	Maximum
*cb*	196.700	96.560	0.491	1.960	397.000
*ud*	211.100	98.190	0.465	6.760	488.700
*dl*	290.100	117.300	0.404	7.580	462.200
*flt*	0.316	0.163	0.516	0.034	0.911
*fml*	0.897	0.105	0.117	0.055	1.036
*hqad*	0.158	0.103	0.652	0.078	0.670

**Table 2:** Results of main effects.

Effect	Coefficient	Standard Error	Z Value	*p* Value	95% Confidence Interval	
*dfi → hqad*	0.284	0.053	5.370	0.000	0.181	0.388
*cb →* *dfi*	0.977	0.008	126.310	0.000	0.961	0.992
*constant*	5.772	0.284	20.340	0.000	5.215	6.328
*ud →* *dfi*	0.960	0.009	112.800	0.000	0.943	0.977
*constant*	7.626	0.355	21.470	0.000	6.930	8.323
*dl →* *dfi*	0.836	0.018	45.610	0.000	0.800	0.872
*constant*	7.547	0.347	21.740	0.000	6.867	8.228

**Table 3:** Results of mediating effects.

Effect	Coefficients	Standard Error	Z Value	*p* Value	95% Confidence Interval	
*dfi →* *flt*	s0.492	0.045	10.930	0.000	0.404	0.580
*constant*	1.932	0.098	19.760	0.000	1.740	2.123
*dfi* *→* *fml*	0.104	0.058	1.790	0.073	−0.010	0.217
*constant*	5.212	0.220	23.640	0.000	4.780	5.644
*flt →* *hqad*	0.642	0.042	15.370	0.000	0.560	0.724
*fml →* *hqad*	0.223	0.043	5.210	0.000	0.139	0.307
*dfi →* *hqad*	−0.054	0.051	−1.060	0.288	−0.153	0.046
*constant*	−0.874	0.238	−3.670	0.000	−1.340	−0.407
*cb →* *dfi*	0.971	0.008	123.710	0.000	0.955	0.986
*constant*	6.198	0.260	23.880	0.000	5.689	6.707
*ud →* *dfi*	0.966	0.008	118.950	0.000	0.950	0.982
*constant*	8.045	0.333	24.130	0.000	7.392	8.699
*dl →* *dfi*	0.836	0.018	45.300	0.000	0.799	0.872
*constant*	7.912	0.328	24.110	0.000	7.269	8.555

**Table 4:** Tests of mediating effects.

Effect	Coefficient	Standard Error	Z Value	*p* Value	95% Confidence Interval	
*dfi → flt → hqad*	0.316	0.038	8.350	0.000	0.242	0.390
*dfi → fml → hqad*	0.023	0.014	1.690	0.090	−0.004	0.050

**Table 5:** Difference analysis of mediating effects.

Effect	Coefficient	Standard Error	Z Value	*p* Value	95% Confidence Interval	
Difference analysis tests	0.293	0.041	7.160	0.000	0.213	0.373

## Data Availability

Data are available from authors upon reasonable request.
